# Nanoscaled alloy formation from self-assembled elemental Co nanoparticles on top of Pt films

**DOI:** 10.3762/bjnano.2.51

**Published:** 2011-08-23

**Authors:** Luyang Han, Ulf Wiedwald, Johannes Biskupek, Kai Fauth, Ute Kaiser, Paul Ziemann

**Affiliations:** 1 Institut für Festkörperphysik, Universität Ulm, Albert-Einstein-Allee 11, 89069 Ulm, Germany; 2 Materialwissenschaftliche Elektronenmikroskopie, Universität Ulm, Albert-Einstein-Allee 11, 89069 Ulm, Germany,; 3 Experimentelle Physik IV, Universität Würzburg, Am Hubland, 97074 Würzburg, Germany

**Keywords:** alloy, Co, CoPt, epitaxy, HRTEM, magnetometry, nanoparticles, Pt, XMCD

## Abstract

The thermally activated formation of nanoscale CoPt alloys was investigated, after deposition of self-assembled Co nanoparticles on textured Pt(111) and epitaxial Pt(100) films on MgO(100) and SrTiO_3_(100) substrates, respectively. For this purpose, metallic Co nanoparticles (diameter 7 nm) were prepared with a spacing of 100 nm by deposition of precursor-loaded reverse micelles, subsequent plasma etching and reduction on flat Pt surfaces. The samples were then annealed at successively higher temperatures under a H_2_ atmosphere, and the resulting variations of their structure, morphology and magnetic properties were characterized. We observed pronounced differences in the diffusion and alloying of Co nanoparticles on Pt films with different orientations and microstructures. On textured Pt(111) films exhibiting grain sizes (20–30 nm) smaller than the particle spacing (100 nm), the formation of local nanoalloys at the surface is strongly suppressed and Co incorporation into the film via grain boundaries is favoured. In contrast, due to the absence of grain boundaries on high quality epitaxial Pt(100) films with micron-sized grains, local alloying at the film surface was established. Signatures of alloy formation were evident from magnetic investigations. Upon annealing to temperatures up to 380 °C, we found an increase both of the coercive field and of the Co orbital magnetic moment, indicating the formation of a CoPt phase with strongly increased magnetic anisotropy compared to pure Co. At higher temperatures, however, the Co atoms diffuse into a nearby surface region where Pt-rich compounds are formed, as shown by element-specific microscopy.

## Introduction

Magnetic nanoparticles (NPs), with narrow distributions of their size and mutual spacing, offer a high potential with respect to both, fundamental and applied studies [1–4]. Although a broad palette of methods has been established for the preparation of such NPs, if additionally their deposition onto a specific substrate in the form of *ordered* arrays over reasonably large areas is required, then the number of applicable fabrication recipes dramatically decreases. Focusing on NP sizes below 15 nm and excluding purely sequential procedures such as those based on scanning probe microscopy techniques [5], one is left with processes relying on the self-assembly of colloids or micelles [6–8]. In the context of magnetic NPs, two prominent examples, both dealing with the preparation of magnetically attractive FePt NPs, which successfully demonstrated fulfillment of the above requirements were presented by Sun et al. applying colloidal chemistry [9] and Ethirajan et al. using micellar methods [10]. Due to the higher variability of the micellar approach with respect to the interparticle distance, this technique has been continually improved and also extended to CoPt NPs as summarized in a recent publication [11]. Despite these successful attempts at fabricating arrays of the specific binary alloy NPs FePt and CoPt, from empirical evidence it appears much easier to prepare elemental NPs along these approaches, as judged from the sheer number of different magnetic or non-magnetic NPs reported. This leads to the simple idea of deposition of an ordered array of elemental NPs onto a metallic film in a first step, and the subsequent reaction of these primary NPs with the subjacent film by temperature-driven alloying. In the case of a reasonable separation of primary NPs, a local binary alloy might form on the nanoscale and maintain the initial particle center-to-center distance. Besides giving insight into nanoalloy formation, such experiments also open the perspective to locally create more complex systems by depositing the NPs on top of pre-alloyed binary or ternary films. Pertinent questions regarding such an approach are: To what extent can the resulting alloy really be confined on the nanoscale; can the orientation of the finally obtained local alloy be controlled by the primary orientation of the film; and how do the resulting phases compare to equilibrium phase diagrams [12]. This last point is closely related to the property changes of the alloy particles considered, in the context of catalysis, such as a narrowing of miscibility gaps upon size reduction [13].

In this paper, the basic idea outlined above is tested by the deposition of hexagonally ordered arrays of Co NPs on top of textured and epitaxial Pt films. Similarly to the previous research interest in FePt equiatomic alloys in the chemically ordered L1_0_ phase, our interest in this system is motivated by the magnetic properties of CoPt alloys exhibiting very large magnetocrystalline anisotropy energy density (MAE) and, directly related to that, a high value of the coercive field *H*_C_ in the direction of the easy axis of magnetization. However, as it has been reported previously, laterally extended CoPt alloy systems may form CoPt_3_ as well [14]. At this composition the MAE is significantly lower than for CoPt in the L1_0_ phase.

The Co volume fraction in our specimens typically amounts to few parts per thousand or less. Therefore, a thorough structural characterization of the alloy formation with standard laboratory equipment is not practical. Instead, we probe the magnetic signatures of alloy formation by X-ray absorption spectroscopy and SQUID magnetometry. The excellent sensitivity of SQUID magnetometers can be exploited, at suitably selected temperatures, to detect the magnetic response corresponding to the Co particles and nanoscale alloys. X-ray magnetic circular dichroism (XMCD) derives its sensitivity from being both element specific and surface sensitive. It is therefore ideally suited for the kind of specimens studied here. In addition to the information contained in (both, SQUID and XMCD) hysteresis loops, we obtain spectroscopic signatures of the average magnetocrystalline anisotropy through the determination of the orbital contribution µ_L_ to the Co magnetic moments [15]. Notable differences of this quantity are known between Co and CoPt alloys [16–17], owing to both Co–Pt hybridisation and atomic structure.

## Results and Discussion

The thermal reaction of metallic NPs with a subjacent metallic film demands the following experimental sequence: 1) Deposition of a thin metal film A exhibiting high quality with respect to grain size, orientation and roughness. 2) Placement of metallic NPs of type B on top of film A. 3) Thermal reaction of A and B and the characterization of the resulting local alloy. In this paper we report the experimental details and results for the specific case of Co NPs on top of Pt(111)/MgO(100) and Pt(100)/SrTiO_3_(100). (For the sake of clarity and brevity, SrTiO_3_(100) is renamed STO(100) in the following).

### Pt films on MgO(100) and STO(100)

Due to the attractive catalytic properties of Pt on top of ceramic supports, much work has been dedicated to the identification of active sites on its surface. For this purpose the controlled growth of Pt films on various single crystalline metal oxides, such as MgO(100) or STO(100), is advantageous. On the resulting epitaxial films, for instance, kink and step sites, with their selective catalytic activities, can be distinguished [13]. Pt films have typically been prepared by sputtering. With regards to the deposition on MgO(100) and STO(100), it is generally agreed that high quality epitaxial Pt(100) films can be obtained with elevated substrate temperature *T*_S_ during deposition. At *T*_S_ = 600 °C epitaxial growth was obtained on MgO(100) or STO(100) substrates [18–20], whereas deposition at ambient temperature led to textured growth of Pt films. Pulsed laser deposition (PLD) produced a similar result for the Pt orientation on MgO(100), at *T*_S_ ≥ 600 °C [21]. The same authors also found a three-dimensional mosaic like island growth under these conditions. In the present study this observation is confirmed by our own PLD experiments performed at *T*_S_ = 600 °C. For sputtering as well as for PLD a switching of the Pt orientation towards (111) orientation has been demonstrated upon lowering of the deposition temperature. This is corroborated by our own PLD experiments. Furthermore, as revealed by AFM measurements, such (111) oriented Pt films exhibit significantly lower roughness on the micron length scale (typical RMS values of 1–2 nm) enabling homogeneous deposition of NPs over the entire sample surface. Despite the island growth mode of Pt(100) when deposited at elevated temperature and the resulting increased roughness (cf. Figure 2), each single island has an almost atomically flat surface (RMS roughness of 0.3 nm).

In the preparation of such Pt films, the following PLD conditions were used: An ArF excimer laser (193 nm, pulse duration 20 ns, 10 Hz repetition frequency) served as the light source for hitting the polycrystalline Pt target. The ablated Pt material was collected on 10 × 5 mm^2^ MgO(100) or STO(100) substrates fixed at a distance of 30 mm from the target. To reduce particulate formation, the target was rotated as well as periodically tilted during the ablation process. To allow calibration of the deposition rate, a movable quartz crystal monitor can be placed at exactly the substrate position. More details on the PLD apparatus, including its UHV chamber, are given in [22–23]. By monitoring the deposition rate as a function of the laser power, an ablation threshold of 2.5 J/cm^2^ was determined for Pt. Standard deposition was performed at 5 J/cm^2^ resulting in a Pt deposition rate of 1 nm/min.

**Figure 1:**
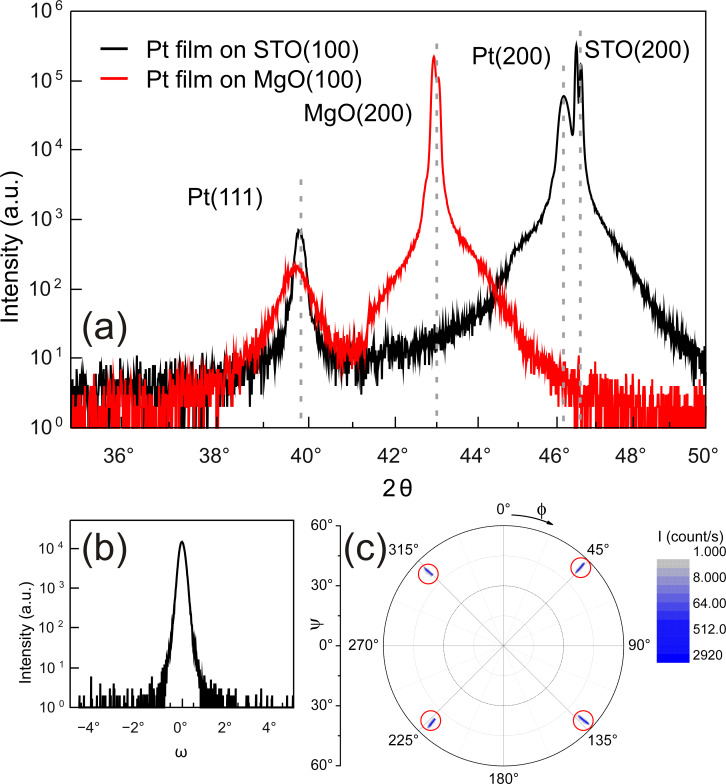
(a) XRD of Pt films on STO(100) and MgO(100) in Bragg–Brentano geometry. The diffractograms clearly show the two different orientations of Pt films when deposited on MgO(100) at ambient temperature or STO(100) at 600 °C. Panel (b) shows the rocking curve on the Pt(200) peak of Pt/STO(100). Panel (c) displays the Pt/STO(100) pole figure measured on the Pt(111) peak position. Diffraction peaks are highlighted by red circles. Details are given in the text.

**Figure 2:**
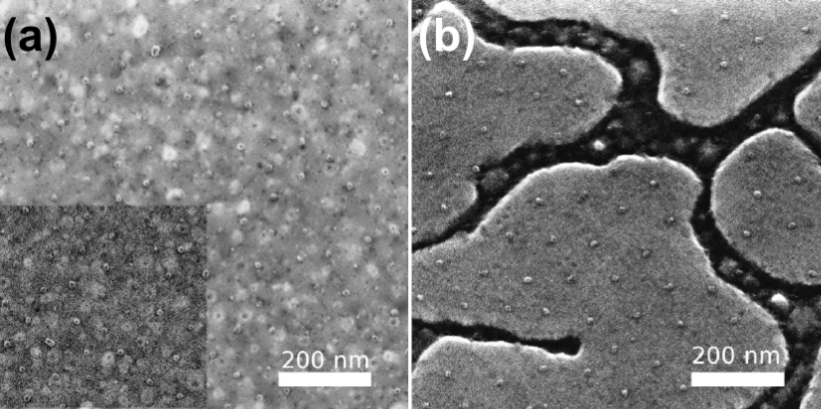
SEM images of Co NPs on Pt(111)/MgO(100) and Pt(100)/STO(100) are displayed in panels (a) and (b), respectively. Co particles are oxidized due to ex-situ transport. For better contrast of NPs three quarters of panel (a) is strongly filtered while in the lower left quarter the original SEM image is displayed.

Standard X-ray diffraction (XRD) diffractograms (Cu Kα radiation, λ = 0.15418 nm) from Pt films deposited on MgO(100) at ambient temperature (nominal thickness 15 nm) and on STO(100) at 600 °C (nominal thickness 40 nm), are presented in Figure 1a. Besides the MgO(200) substrate peak, the diffractogram of the film deposited at ambient temperature exclusively reveals the Pt(111) peak as expected. The rocking curve on the Pt(111) peak has a full width at half maximum (FWHM) of 14.4° indicating a rather poor degree of (111) orientation. Pole figure scans reveal a practically random in-plane orientation of the Pt(111) film on MgO(100) deposited at ambient temperature (not shown). The grain size was estimated using Scherrer’s formula to be about 16 nm, which is in good agreement with the nominal film thickness. For comparison, the in-plane dimension of the grains is about 20–30 nm, as determined from scanning electron microscopy (SEM) images (cf. Figure 2a). XRD from the Pt film on STO(100) deposited at 600 °C reveals two orientations: First, the Pt(111) orientation is present but with a much larger grain size as indicated by the sharper peak. More important is the observation of the Pt(200) peak slightly above 46° having a much higher diffraction intensity. Evaluation of the intensity ratio of the Pt(200) and Pt(111) peaks yields *I*_(200)_/*I*_(111)_ = 86 and, moreover, using tabulated powder diffraction intensities, one finds an intensity ratio of *I*_(200)_/*I*_(111)_ = 0.53 for Pt powder. Thus, the Pt film deposited at elevated temperature has predominantly the Pt(100) orientation on STO(100). Qualitatively similar results were also obtained for Pt films on MgO(100) when deposited above 600 °C (not shown), albeit with a lesser degree of Pt(100) orientation. Figure 1b presents the rocking curve on the Pt(200) peak of the Pt film on STO(100). The small rocking width of FWHM = 0.29° indicates a high degree of orientation of the film. To test possible epitaxy of this film a pole figure was measured at the Pt(111) peak position (2θ = 39.8°) by scanning both the in-plane angle Φ and the tilting angle ψ. Figure 1c presents the result in a polar plot. Four (111) peaks are observed at ψ = 54.7° and Φ = 45°, 135°, 225°, and 315°. Note that a slit aperture was used here to reduce the acquisition time, leading to a broadening of the diffractogram in ψ direction. From the above diffraction peaks and the known orientation of the STO substrates we find a cube-on-cube growth of the Pt film on the STO(100) with orientations Pt(100)||STO(100) and Pt[010]||STO[010].

### Co nanoparticles on Pt films

The preparation of metal NPs is based on spherical reverse micelles formed by the diblock copolymer poly(styrene)[*m*]-*block*-poly(2-vinylpyridine)[*n*] (PS[*m*]-*b*-P2VP[*n*]) in toluene (*m*, *n* indicate the number of monomers for each block, and, thus, determine the size of the resulting micelles). In this case, the hydrophobic PS forms the outer corona of the micelles and the hydrophilic P2VP their core. To this core, metal precursors can be selectively bonded, and thus the micelles serve as carriers for these precursors, during their own self-assembly, when deposited onto a substrate. The standard way to accomplish such a deposition is optimized dip coating, which leads to a single monolayer of hexagonally ordered micelles. In the next step, the organic constituents are completely removed by exposure to oxygen plasma, while simultaneously the precursor material is nucleated into a metal or metal oxide NP, without losing the original hexagonal ordering. In case of oxide NPs, an additional treatment in hydrogen plasma finally delivers the desired hexagonally ordered array of metal oxide NPs. More details on this fabrication process can be found in [11,24–25].

For the preparation of Co NPs for the present study, PS[1779]-*b*-P2VP[857] diblock copolymers were employed in combination with anhydrous CoCl_2_ as precursor at a loading rate of *L*_Co_ = 0.5 (*L*_Co_ is defined as the ratio of ligated Co within the micellar core to the total number of pyridine moieties). The two parameters *L*_Co_ and (*n* + *m*), together with the substrate velocity during dip coating (15 mm/min), determine the particle size and interparticle distance. In the present study, these parameters were fixed as given above resulting in Co NPs with diameters of about 7 nm and mutual separation of 100 nm. More details on the specific preparation and chemical control of the final NP arrays are presented in reference [11].

It should be noted that, although the fabrication is highly reproducible for a given micellar solution, separately prepared solutions from the same commercial copolymer may nevertheless deliver a different size distribution of the formed micelles, despite filtering. For that reason, in the present study samples were prepared in parallel from a single solution in order to guarantee arrays of NPs with reproducible size and spacing, before starting the various annealing experiments. All NP arrays were examined by SEM to determine the interparticle distance, lateral diameter and degree of hexagonal order. In the following we describe NPs as being “in the as-prepared state”, meaning that a 10 min reduction process was applied, in hydrogen plasma at 10^−1^ mbar at *T* = 200–250 °C, to reliably restore the pure metallic state after the inevitable ex-situ transfer. Similarly, the thermal reaction of the Co NPs with the subjacent Pt film was induced by heating to a given temperature for 30 min in the presence of 10^−4^ mbar H_2_ to avoid any oxidation.

The results corresponding to the above experimental steps are described below. The SEM image (Hitachi S5200) in Figure 2a shows the in-plane grains of a typical Pt(111) film, with an average size of approximately 20–30 nm and a RMS roughness below 2 nm as determined by AFM. On top of the Pt(111) film Co NPs can be observed. Note that strong image filtering was applied here to better visualize the NPs on the Pt(111) film, and the lower left section shows part of the original SEM image. Figure 2b illustrates the arrangement of Co NPs on top of a 50 nm epitaxial Pt(100) film. Co NPs form hexagonal arrays on the micron-sized islands. The islands are single crystalline (cf. Figure 1) flat surfaces, with only a few atomic steps, and a RMS roughness of 0.3 nm (AFM). The darker areas consist of smaller Pt grains at a reduced height compared to the islands. The films, however, are continuous at the film–substrate interface and possess metallic conductivity. The fraction of height-reduced areas depends on the film thickness and is below 10% for nominally 50 nm Pt(100) films.

### Effects of annealing on Co nanoparticles on Pt(111) films

**Figure 3:**
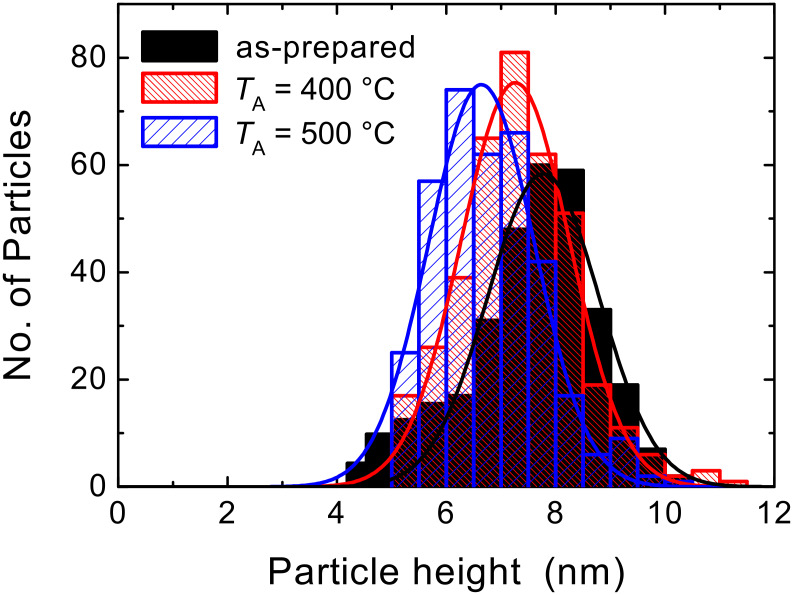
AFM height distributions of Co NPs on Pt(111)/MgO(100) in the as-prepared state and after annealing at *T*_A_ = 400 °C and 500 °C for 30 min. Additionally, the Gaussian fits to the measured size distributions are shown. Note that the particles are oxidized when examined ex-situ by AFM.

AFM measurements were performed on Co NPs on Pt(111) to corroborate changes of the vertical height of the Co NPs, as well as to monitor the effect of increasing temperatures on this height. Here, the same sample was successively annealed at increasing temperature under a H_2_ atmosphere at 10^−4^ mbar. AFM measurements were performed ex situ, consequently the NPs oxidized in the ambient air. After inspection the specimen was reduced in hydrogen plasma before the next annealing step was applied. This procedure guarantees that the NPs as well as the film are always metallic during the annealing process. Due to the limited in-plane resolution of AFM, particle sizes are characterized by the maximum height with respect to substrate plane. Such height distributions obtained for the as-prepared NPs, as well as after annealing at 400 °C and 500 °C, are given in Figure 3. Each annealing step resulted in a reduction of the average particle height. This decrease may arise from different processes, such as deformation due to increased substrate wetting, loss of Co atoms due to evaporation and bulk diffusion, or a combination of these processes. While the possibility of metal NPs wetting the metal substrate [26] is not excluded in this study, the TEM investigation (see below) clearly reveals a spherical particle shape before annealing and subsequent vanishing of particles after annealing (cf. Figure 7), favoring the model of Co atom loss. To estimate the degree of Co atom loss, we calculate the corresponding metallic NP diameters by assuming the formation of CoO with a lower density of 6.44 g/cm^3^ compared to the density of metallic Co, at 8.90 g/cm^3^, in the bulk. Assuming spherical particles, this estimate leads to mean heights of pure Co NPs of 7.0 nm, 6.5 nm, and 6.0 nm in the as-prepared state and after annealing at an annealing temperature *T*_A_ = 400 °C and *T*_A_ = 500 °C, respectively. The mean height reduction from 7 nm to 6 nm yields a 37% loss of Co from the NPs after annealing at 500 °C for 30 min.

Consequently, a significant amount of the NP material is no longer discernable by AFM. A simple explanation attributing this decreasing particle size to thermal evaporation caused by vapor pressure enhancement due to the reduced size of the NPs is, however, unlikely for 7 nm Co NPs at an *T*_A_ below 500 °C. To further clarify the processes involved, we additionally carried out a surface and element specific characterization of the specimen by in-situ X-ray photoelectron spectroscopy (XPS). These data (not shown) clearly demonstrate that the intensity ratio of the Co 2p peaks with respect to the Pt 4f peaks decreases by about 40% after annealing at 500 °C, compared to the as-prepared state. This change is in good agreement with the results from AFM inspection above. Since XPS samples the surface region of the Pt film, including the Co NPs, we can conclude that the observed “AFM loss” cannot be attributed to Co atoms still remaining within the probing depth of XPS, which is restricted by a photoelectron mean free path of about 1.6 nm [27]. Rather, diffusion along the large number of grain boundaries in the Pt(111) film on MgO(100) is expected. On Pt(100) films with micron-sized, atomically-flat surfaces this diffusion channel does not exist for most of the NPs, thus markedly different diffusion and alloying behavior is expected. An additional AFM inspection of the Co NPs on Pt(100) after annealing at 500 °C for 30 min (not shown) yielded an average particle height of 5.4 nm, which is significantly smaller than the finding on the Pt(111) film (cf. Figure 3). By ex-situ AFM measurements, however, we cannot distinguish different modes of diffusion on Pt(100) and Pt(111) films. In the context of the following magnetic measurements and HRTEM investigations, this point is discussed in more detail.

### Magnetic properties of Co NPs on Pt(100) and Pt(111) films

**Figure 4:**
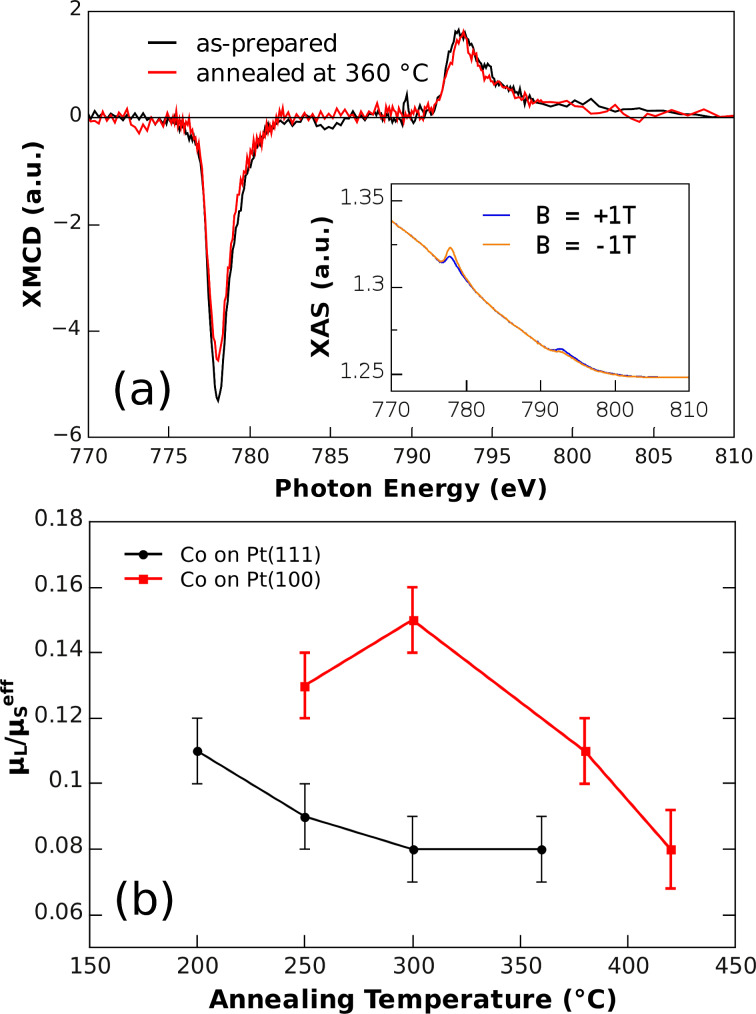
Panel (a) shows XMCD difference spectra for Co NPs on Pt(111) in the as-prepared state and after annealing at 360 °C for 30 min (final state). The inset displays the absorption spectra for external fields of µ_0_H = ± 1T in the as-prepared state. Panel (b) presents the resulting ratios of orbital-to-spin moments for Co NPs on Pt(111) and Pt(100) films as a function of annealing temperature T_A_ (holding time 30 min). The lines are given as guides to the eye.

Co L_3,2_ XMCD measurements were made on specimens of the deposited Co NPs on both textured Pt(111) and epitaxial Pt(100) films, as function of annealing temperature. The investigations were performed at the bending magnet beamline PM3 of BESSY II synchrotron radiation facility at the Helmholtz-Center Berlin, Germany. Throughout all steps of the specimen investigation, ultrahigh vacuum conditions were maintained, except for the annealing steps carried out in a H_2_ atmosphere at 10^−4^ mbar. All XMCD measurements were taken at low temperature (T ≈ 12 K) and at normal incidence of the circularly polarized X-rays (p ≈ 0.93), by recording the sample drain current (total electron yield, TEY) as a function of photon energy. External fields of up to µ_0_*H* = ± 3 T were available. Spectra and hysteresis loops were recorded and evaluated by methods described previously [11,28–30]. The insert to Figure 4a displays a typical pair of XAS energy scans, obtained in applied fields of µ_0_*H* = ± 1 T, sufficient to achieve magnetic saturation. While the Co L_3,2_ resonances as well as the magnetic dichroism are clearly visible, we note that even the resonant Co signal amounts to only a fraction (≈1%) of the strong TEY background (≈170 pA) generated in the Pt film. In addition, because the background is curved, a quantitative determination of the (spin and orbital) magnetic moments from the XMCD sum rules is problematic. We will therefore resort to the more robust procedure of evaluating the ratio of the orbital magnetic moment to the effective spin magnetic moment µ_L_/µ_S_^eff^, where the effective spin moment µ_S_^eff^ = µ_S_ + 7 µ_T_ contains two contributions: The spin moment µ_S_, as well as the magnetic dipole moment µ_T_, which relates to the anisotropy of the spin density distribution. As the magnetic dipole term may be quite significant in CoPt systems due to the structural anisotropy in the chemically ordered L1_0_ phase [31], only µ_S_^eff^ will be discussed for the NPs in this study.

Figure 4a compares two XMCD difference spectra, i.e., the difference in the X-ray absorption spectra, for antiparallel external fields (here µ_0_*H* = ± 1 T) collinear to the incident X-ray beam, of Co NPs deposited on Pt(111) in the as-prepared state and after annealing at *T* = 360 °C for 30 min. Both spectra are scaled to the same L_2_ dichroic amplitude at about ≈794 eV. This scaling facilitates the comparison of the orbital magnetic moment, which now correlates with the resulting L_3_ dichroic intensity. Details are discussed further, below.

For Co NPs on Pt(111) films, XMCD spectra were measured for as-prepared and annealed samples. Increasing annealing temperatures (250 °C, 300 °C, 360 °C) were used, with the samples held for 30 min at each temperature. The results reveal a monotonic, small decrease of µ_L_/µ_S_^eff^ for increasing annealing temperatures approaching µ_L_/µ_S_^eff^ = 0.08 at *T*_A_ = 300 °C and above (Figure 4b). Although the comparison to isotropically averaged values of µ_L_/µ_S_^eff^ = 0.095 for Co [32], µ_L_/µ_S_^eff^ = 0.09 CoPt [16], and µ_L_/µ_S_^eff^ = 0.15 for CoPt_3_ [17] in bulk samples or thin films is generally useful, one has to consider that these values may vary due to size effects in NPs, such as enhanced surface moments. Thus, we restrict ourselves to the direct comparison of annealing effects on Pt(111) and Pt(100) films. On Pt(111), huge orbital moments, as observed for Co adatoms on Pt(111) single crystals [33], were not found. Moreover, the AFM size distribution discussed above (cf. Figure 3) only showed a slight reduction of the metal particle height from about 7 nm to 6.5 nm after annealing at *T*_A_ = 400 °C. Our finding, by XPS, of a simultaneously reduced Co content after annealing suggests that Co atoms diffuse away from the surface along grain boundaries at elevated temperature. Thus, we speculate that after annealing the size-reduced NPs on Pt(111) remain in a pure Co, or at least Co-rich, phase having a rather low orbital moment. Nevertheless, we note that Co atoms generally possess larger spin moments in CoPt alloys (1.76 µ_B_ per atom for L1_0_ ordered CoPt alloy [16] and 1.60 µ_B_ per atom for L1_2_ ordered CoPt_3_ alloy [17]) compared to pure Co (1.55 µ_B_ per atom [28]). Thus, an increase of the spin moment of up to 15% can be expected. Surface alloy formation therefore might additionally contribute to the reduction of the ratio µ_L_/µ_S_^eff^.

For Co particles deposited onto the large islands of the epitaxial Pt(100) film (cf. Figure 2b) the situation is quite different. Starting from µ_L_/µ_S_^eff^ = 0.13 we found an initial increase to µ_L_/µ_S_^eff^ = 0.15 after annealing at 300 °C for 30 min. At higher annealing temperatures µ_L_/µ_S_^eff^ decreased, and after annealing at *T*_A_ = 420 °C a similar value to that for the Co NPs on Pt(111) was observed. Although alloying results in slightly increased spin moments, pointing to lower ratios µ_L_/µ_S_^eff^, the initial increase in our experiments can only be explained by a faster growth of the orbital moment upon annealing. Such rising orbital moments signal alloy formation on the Pt(100) surface.

If such an alloy formation preserves the (100) starting orientation of the film, one expects the easy axis of magnetization and, thus, the largest orbital moment of resulting chemically ordered CoPt thin films [16] or Co/Pt multilayers [34] to be perpendicular to the Pt atomic layers. Indeed, within the error bars, the observed maximum of µ_L_/µ_S_^eff^ = 0.15 is found rising towards the expectations for both, ordered CoPt (µ_L_/µ_S_^eff^ = 0.16) and CoPt_3_ alloys (µ_L_/µ_S_^eff^ = 0.19) in the easy axis of magnetization, corroborating the idea of alloy formation at these intermediate annealing temperatures. Such a finding is comparable to results from ultrathin Co films deposited on Pt(100) [35] and Pt(111) [36] single crystal surfaces, where alloying occurs between 300 °C to 400 °C. An alignment of the easy axis of magnetization should, however, be visible in the hysteresis loops discussed below.

**Figure 5:**
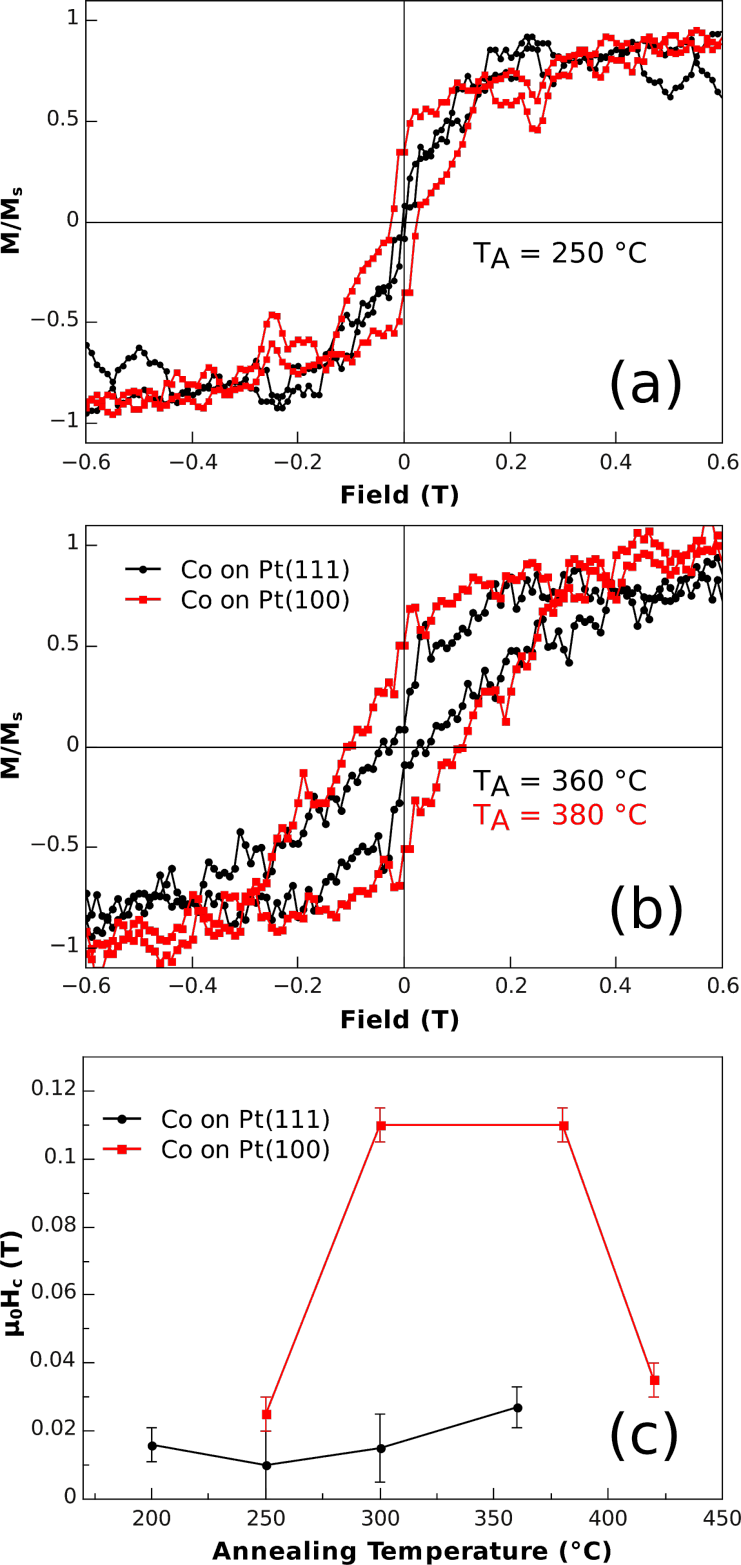
Element specific XMCD hysteresis loops measured at the Co L_3_ maximum dichroic signal at T = 12 K and out-of-plane geometry for Co NPs on Pt(100) and Pt(111) films after annealing at (a) *T*_A_ = 250 °C and (b) 360 °C on Pt(111) and 380 °C on Pt(100). Panel (c) shows the evolution of the coercive field as function of the annealing temperature (holding time at each *T*_A_: 30 min). The lines are given as guides to the eye.

Obviously, two competing effects play a decisive role in the present study, i.e., diffusion and the formation of local surface alloys. Insight into the progress of these processes can be provided by the measurement of hysteresis loops after the various annealing steps. In Figure 5 element specific XMCD hysteresis loops, measured along the surface normal, are displayed for two temperatures of the annealing series. Although the hysteresis loops appear quite noisy due to the low volume fraction of magnetic material on the surface, the remnant magnetization, coercive fields and the shape of the hysteresis are sufficiently well defined to allow the confirmation of alloy formation for Co NPs on Pt(100) films.

After the first annealing step (*T*_A_ = 250 °C) a narrow hysteresis loop was observed with a coercive field of µ_0_*H*_C_ = 27 mT on Pt(100) films, while no clear opening was detected for Co NPs on Pt(111), within the experimental uncertainties. The largest difference of coercive fields was obtained after annealing in the interval *T*_A_ = 300–380 °C for 30 min, as can been seen in Figure 5c. Whereas for Co NPs on the Pt(111) film only a slight increase up to about µ_0_*H*_C_ = 27 mT was measured, the evolution of the coercive field on Pt(100) epitaxial films was more pronounced. For *T*_A_ = 300 °C it jumped to 110 mT, which is comparable to previous reports on Co_0.25_Pt_0.75_ films [37]. Thus, a significant difference for the two types of Pt films is observed, which parallels the changes of the ratio of orbital-to-spin moments discussed above. At still higher temperatures (*T*_A_ = 420 °C), however, the *H*_C_ enhancement is followed by a pronounced *H*_C_ reduction for Co NPs on Pt(100). Similarly, the two types of Pt films exhibit a clear difference in their remnant magnetization *M*_R_. After annealing the Co NP on Pt(111) at 360 °C, *M*_R_ found at 12 K was rather low and hardly detectable due to the small signals, whereas after annealing Co NP on Pt(100) at 380 °C (Figure 5b) *M*_R_ was about 0.5·*M*_S_ (*M*_S_: saturation magnetization). The higher the value of *M*_R_, the larger the number of magnetic entities found aligned in the direction of measurement. For a preferred structural orientation of NPs with respect to the Pt(100) film, however, *M*_R_ is too low, it actually matches well the value for Stoner–Wohlfarth particles with random orientation of the anisotropy axis. Here one may speculate that much longer annealing times at an *T*_A_ of around 350 °C could lead to at least some structural orientation relative to the Pt(100) film [38]. In summary, the hysteresis loops in perpendicular orientation reveal no dramatic changes of coercive fields for the Co NPs on the Pt(111) film, whereas on the Pt(100) film µ_0_*H*_C_ = 110 mT is more than twice as large as the value found for metallic Co nanoparticles of comparable size after application of a similar sample treatment [39]. This finding additionally confirms the lateral spread of Co atoms.

**Figure 6:**
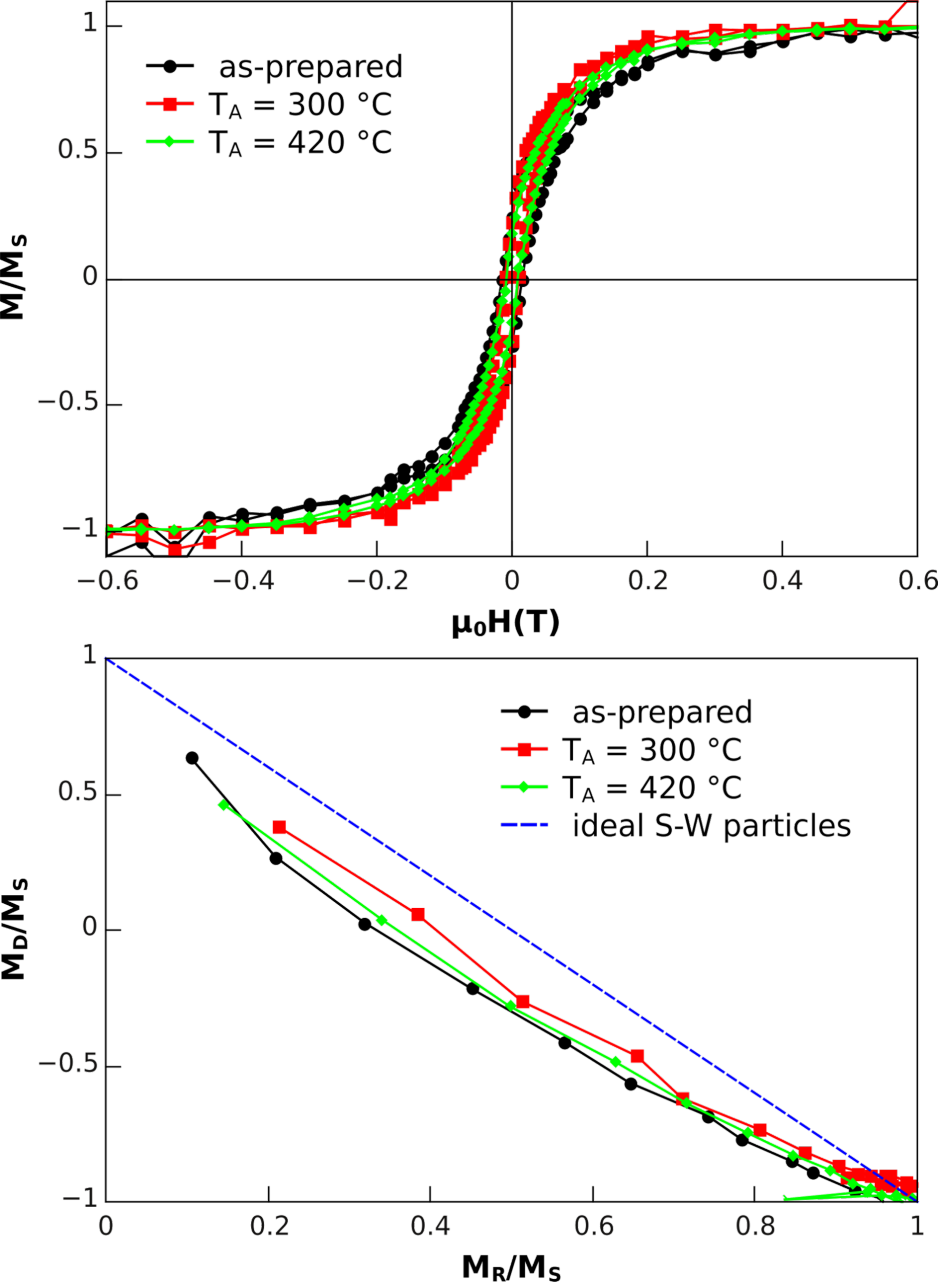
(a) In-plane hysteresis loops measured by SQUID magnetometry at *T* = 29 K, i.e., close to the compensation temperature of the diamagnetic MgO substrate and the paramagnetic Pt(111) film on top. In (b), the Henkel plots for three annealing steps are presented. The data of the as-prepared state was taken from [11]. Details are discussed in the text.

Additional in-plane hysteresis loops were measured by SQUID magnetometry for 7 nm Co NPs on Pt(111) films after different annealing steps. Note that each hysteresis loop was measured on a separate sample to exclude any effect of the thin SiO cover layer used for preservation in ambient conditions after in-situ annealing. Contrary to XMCD, SQUID magnetometry measures the total magnetic moment of the sample, i.e., the NPs, the paramagnetic Pt film, the SiO protective layer and the diamagnetic MgO(100) substrate. Usually the magnetic response of the support easily overwhelms the total magnetic moment of the tiny amount of ferromagnetic material in the NPs. In the present system one can benefit from the paramagnetic response of the Pt(111) film and paramagnetic impurities in MgO compensating the diamagnetic signal of the substrate. Since the diamagnetism of MgO is temperature independent and the paramagnetic signal follows Curie’s law at low temperatures [40], compensation can be achieved at an appropriate temperature, which is experimentally determined to be around 29 K for our samples. As the non-ferromagnetic background was strongly reduced, a reasonable signal quality was obtained as shown in Figure 6a, after subtraction of a smaller slope arising from the sum of substrate and film contributions.

The in-plane hysteresis loops shown in Figure 6a after different annealing steps show almost no change. The coercive fields are around µ_0_*H*_C_ = 15 mT and the remanence amounts to *M*_R_/*M*_S_ ≈ 25%. Both values are consistent with the corresponding normal incidence XMCD data (Figure 5). The similarity between the in-plane (SQUID) and out-of-plane (XMCD) hysteresis loops suggests that the Co NPs on Pt(111) are essentially magnetically isotropic and thus posses little MAE. Such finding confirms that CoPt alloys with high MAE have not been formed by the annealing of Co NPs on Pt(111). Additionally, DC-demagnetization (DCD) and isothermal remnant magnetization (IRM) [41–42] were measured for identical external magnetic fields, and the remnant magnetizations after DCD (*M*_D_) and IRM (*M*_R_) yielded the so-called Henkel plot [43] shown in Figure 6b. From this plot additional information relating to the possible magnetic interaction among NPs can be obtained. In the case of non-interacting ideal Stoner–Wohlfarth (S–W) NPs the corresponding Henkel plot is linear with a slope of −2, indicated by the dashed line in Figure 6b. Recently, we have shown that this linear behaviour is obtained for Co NPs on Si/SiO_2_ substrates at *T* = 10 K with interparticle distances comparable to those in the present samples [11]. The experimental curves for different annealing temperatures closely resemble each other and all are found to be near to the Stoner–Wohlfarth line. The small deviation at intermediate demagnetization fields can be understood as the effect of thermal fluctuation at *T* = 29 K [41]. Such a finding implies that there is no significant dipolar or exchange coupling between neighbouring magnetic entities, and the annealing does not lead to agglomeration of Co atoms, although significant diffusion of Co atoms is expected.

### HRTEM of Co NPs on Pt(100) films

Since the CoPt phases with high MAE were only formed by annealing on epitaxial Pt(100) films, we concentrated our HRTEM investigations on this system. For this study a MgO(100) substrate was used and the Pt film was deposited at 600 °C. Before the TEM investigation a protective layer of SiO_2_ was deposited to prevent NP oxidation. TEM samples were prepared for cross section imaging by standard techniques, namely mechanical grinding and polishing followed by low angle Ar^+^-ion etching. Bright-field TEM and aberration corrected HRTEM images were taken on a FEI Titan TEM equipped with a *C*_s_ imaging corrector. Scanning TEM and energy dispersive X-ray spectra (EDX) were acquired on a FEI Titan equipped with an HAADF-STEM detector and EDAX SiLi X-ray detector.

**Figure 7:**
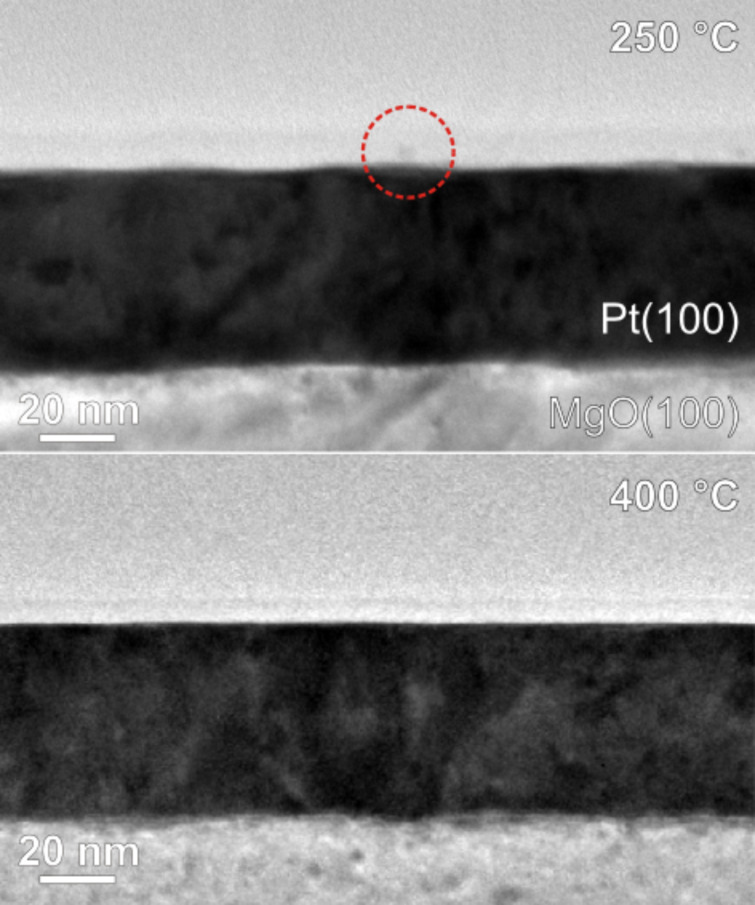
Bright field TEM images of Co NPs on Pt(100) films after annealing at *T*_A_ = 250 °C (as-prepared state) and *T*_A_ = 400 °C.

Typical bright field TEM images in the as-prepared state (*T*_A_ = 250 °C) and after annealing at 400 °C are shown in Figure 7. Apart from the MgO substrate and the Pt(100) film, the protective layer of SiO_2_ is also visible. In the as-prepared state an isolated Co particle could be identified, as indicated by the red circle in the centre of the image. After annealing at 400 °C, however, particles could no longer be detected on the Pt film. This finding was confirmed on three samples at annealing temperatures of 400 °C and above.

**Figure 8:**
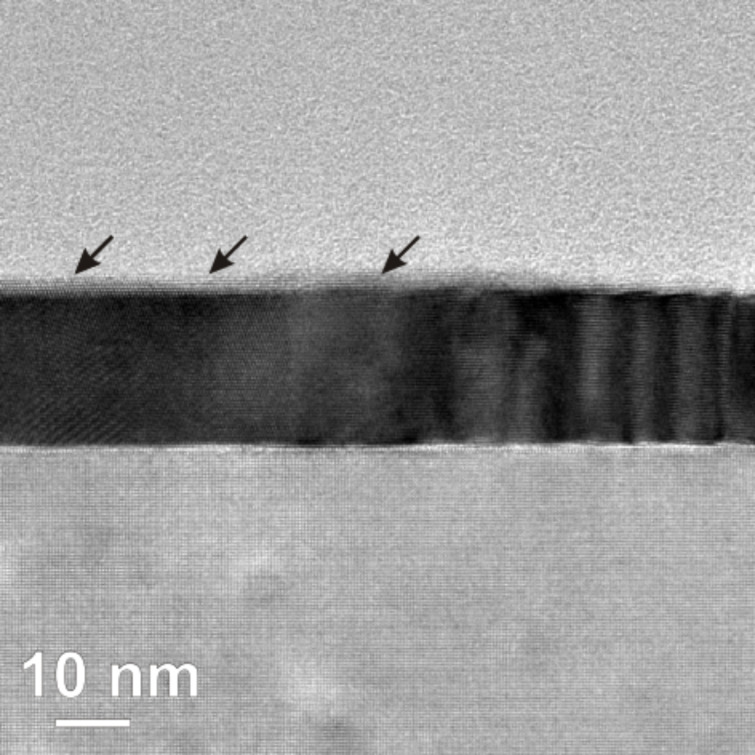
HRTEM image of annealed Co NPs on Pt(100) film after *T*_A_ = 500 °C for 30 min. The arrows indicate a thin surface layer of Pt-rich Co*_x_*Pt_1−_*_x_* alloy on top of the Pt (seen by the weaker absorption contrast). Note that this sample has not been covered by any protective layer.

Additionally, HRTEM investigations were performed for Co NPs annealed at 500 °C, as shown in Figure 8. The structure indicated by the arrows clearly demonstrates alloy formation along the Pt surface. Such structures have typical thickness of 2–3 nm and lateral spread of 30–40 nm. The lighter contrast of such structures indicates that their constituents contain elements with lower atomic number than Pt. This result suggests that the observed structures are local alloys formed by lateral diffusion of Co atoms from the originally spherical Co NPs and simultaneous alloying with the Pt film underneath. Assuming the 7 nm Co NPs are completely transformed into the Co_50_Pt_50_ phase, the resulting volume of the alloy is expected to be about 300 nm^3^ per Co NP. This estimated volume is far too low to account for the observed dimensions of the alloy structure revealed by HRTEM. Thus, the formation of a much more Pt-rich phase is suggested by these images. This conclusion is also consistent with the magnetic investigations indicating that, after annealing above 400 °C on Pt(100) films, the MAE decreases and ratio of orbital-to-spin moment approaches the value of a disordered Pt-rich Co*_x_*Pt_1−_*_x_* alloy.

Although only a chemically disordered Co*_x_*Pt_1−_*_x_* alloy is observed in this sample, an important feature of such a local alloying process can be recognized: The alloy formed at the surface has the same crystalline orientation as the Pt film underneath, as shown in Figure 8. Such an epitaxial relation is potentially very useful for the alignment of the magnetic easy axis if the local alloy has high magneto-crystalline anisotropy energy.

**Figure 9:**
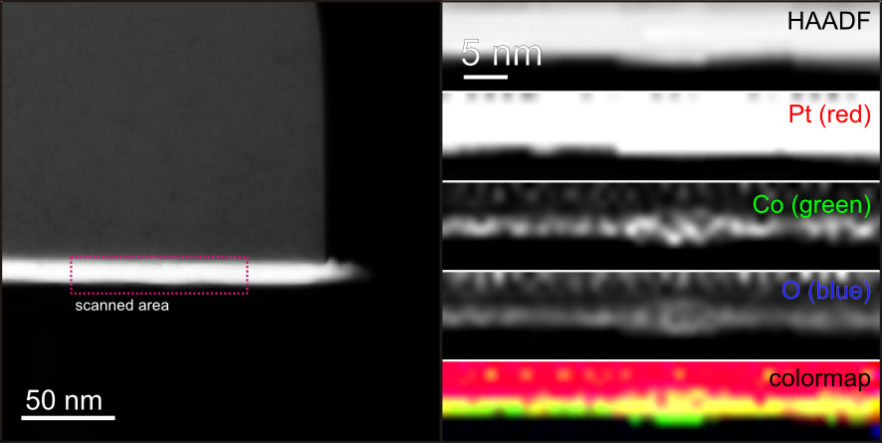
The left image shows the high angle annular dark-field (HAADF) image of the sample shown in Figure 8 using scanning TEM. EDX scanning TEM analysis of the selected area is shown on the right for Pt, Co, and O. A color map is reconstructed by using Pt as red color channel, Co as green color channel, and O as blue color channel. The yellow region at the film surface consists of both, Co and Pt and, thus, indicates alloy formation.

The formation of local alloys is further confirmed by scanning TEM analysis with EDX on the same sample as in Figure 8. The left panel of Figure 9 shows an overview image of the sample, where the bright stripe corresponds to the Pt thin film due to the elemental contrast (contrast scales with *Z*^2^) of the HAADF-STEM detector. Locally resolved EDX-STEM analysis (1 nm scan width, beam diameter ~0.5 nm) was performed in the area indicated by the red box, and the corresponding Pt, Co and O elemental maps are shown on the right. It is evident from the Co elemental map that the Co atoms are distributed along the Pt surface, giving the direct proof of Co surface diffusion. The large agglomeration with higher Co concentration in the center likely corresponds to the initial position of one Co NP. A combined elemental map is also given by mapping Pt, Co, O signals to red, green and blue channels, respectively. Apart from the Pt film (red region) and the residual of the Co NP (green island in the center), the yellow region at the film surface consists of both, Co and Pt. This can be interpreted as the region of alloy formation. It is worthwhile noting that a small concentration of oxygen can also be identified, which essentially follows the distribution of Co atoms. Since this sample has not been covered by any protective layer due to the requirements of the EDX-STEM analysis, oxidation of Co is expected. The EDX-STEM analysis is an additional confirmation of the lateral spread of Co atoms.

## Conclusion

We investigated the thermally driven diffusion and formation of local alloys starting from self-organized metallic Co NPs deposited on top of Pt(100) and Pt(111) films. For this purpose Pt films with (100) and (111) orientations were prepared on MgO(100) and STO(100) substrates by pulsed laser deposition. When deposited at elevated temperature (600 °C and above) epitaxial growth was achieved on STO(100) and MgO(100) with micron-sized atomically flat islands. When the deposition temperature was held at ambient temperature the Pt films exhibited a (111) structure with a lateral grain size of 20–30 nm as estimated by SEM. On these two types of films metallic Co particles (diameter 7 nm) were prepared by a micellar approach and reactive plasma etching, resulting in interparticle distances of about 100 nm. These well separated NPs serve here as local Co reservoirs on the nanoscale. By annealing experiments at various temperatures up to 500 °C, the alloy formation was characterized by various techniques (SEM, AFM, TEM, XPS, XMCD and SQUID magnetometry). All annealing experiments were performed in the pure metallic state, thus excluding any effects of (partial) oxidation of Co NPs and Pt films. In a first survey of local alloy formation we investigated the remaining Co particle height on Pt(111) films by AFM after different annealing steps. Here, a decreasing particle diameter, from 7 nm to 6 nm, was observed after annealing at *T*_A_ = 500 °C for 30 min. This loss of Co material, however, is attributed to diffusion of Co atoms into the subjacent Pt film, as suggested by XPS.

Since the magnetism of metallic Co and various CoPt alloys is known to change strongly due to the huge variations of MAE, and sufficient sensitivity is guaranteed compared to standard structure investigations (e.g., XRD), we investigated the magnetic properties by XMCD and SQUID magnetometry on both Pt(100) and Pt(111) films. On the latter, annealing led to a decreasing ratio of orbital-to-effective spin moment µ_L_/µ_S_^eff^. Moreover, no drastic changes of the coercive field were found perpendicular to the film plane. Additional in-plane measurements by SQUID magnetometry suggest that the shrinking NPs essentially remain in a low anisotropy phase, presumably as pure Co NPs on the surface and Co atoms diffusing along grain boundaries facing a Pt-rich environment. Moreover, magnetic coupling of NPs can be excluded as shown by Henkel plots.

On the Pt(100) epitaxial films a completely different behavior has been observed up to intermediate annealing temperature *T*_A_ = 380 °C. In this regime, both µ_L_/µ_S_^eff^ and the coercive field rise to values exceeding the expectations for pure Co NPs. This finding indicates formation of local Co*_x_*Pt_1−_*_x_* alloys. The exact phase, however, cannot be determined on the basis of our data. At higher T_A_ the magnetic indicators µ_L_/µ_S_^eff^ and *H*_C_ start decreasing, probably matching the experiments on the Pt(111) film at slightly higher *T*_A_ values. The local distribution of Co atoms after annealing at *T*_A_ = 500 °C was imaged by HRTEM and EDX-STEM. At this temperature the observed volume of the Co–Pt solid state reaction is much larger than the initial volume of Co NPs. Although a quantitative statement is not possible here, we can conclude that a Pt-rich Co*_x_*Pt_1−_*_x_* phase has been formed.

**Figure 10:**
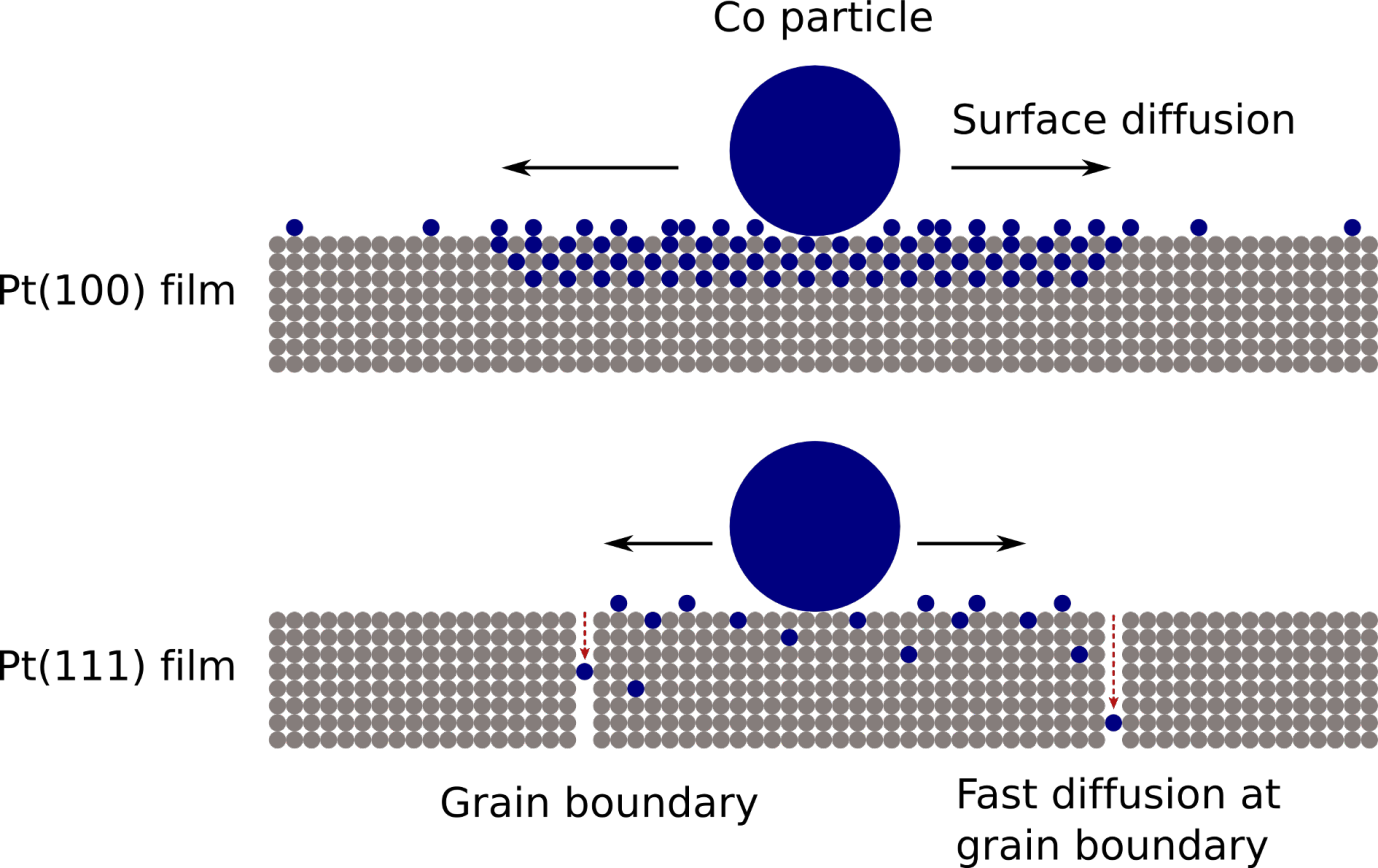
Proposed model of local alloying and diffusion of Co NPs on Pt films. Details are discussed in the text.

The results above lead to the conclusions displayed in Figure 10 for the two systems under investigation. Annealing of Co NPs on Pt(111) films gives rise to surface diffusion of Co atoms. The microstructure of the film consisting of rather small grains (20–30 nm), however, lets the diffusing atoms easily find grain boundaries in the Pt film. It is well-known that the grain boundaries act as fast diffusion channels. Thus the grain boundaries in the Pt(111) film effectively remove Co atoms from the surface. The limited Co surface concentration implies that only disordered Co*_x_*Pt_1−_*_x_* phases with low MAE can be formed in the bulk. As a result, the film microstructure hinders the formation of ordered CoPt alloy with high MAE.

On epitaxial Pt(100) films with micron-sized islands having high structural quality, diffusion along grain boundaries plays a minor role. Consequently, a higher concentration of Co surface atoms can be established in the vicinity of the as-prepared Co NPs, and alloying spreading from the initial NP location becomes possible. At intermediate *T*_A_ the observations strongly suggest a phase with enhanced MAE, as indicated in Figure 10. However, for the chosen *T*_A_ and 30 min annealing time, the detailed composition of this phase cannot unequivocally be determined. Annealing at higher temperature leads to further diffusion of Co into the Pt film. Due to the locally reduced Co concentration, Pt-rich alloys are formed. Nevertheless, the epitaxial orientation of the alloyed region can be clearly identified.

The above findings motivate further investigations at intermediate annealing temperatures for longer periods of time. Under these conditions a local alloy close to Co_50_Pt_50_ with high MAE may form. Additionally, the epitaxial relation to the Pt(100) film underneath could serve as a template to completely align the easy axis of magnetization of the alloy phase perpendicular to the sample plane. Such experiments are currently under way.
